# Chromosome and Plasmid Sequences of *Pantoea* sp. Strain SOD02 Isolated from an Urban Freshwater Stream

**DOI:** 10.1128/mra.00822-22

**Published:** 2022-09-21

**Authors:** Ibrahim Abaherah, Geraldine Almonte, Kathryn Donohue, Daniel Furey, Kathleen Garvey, Jacqueline Joyal, Devyn Luden, Kathryn Mulvey, Sean O’Donnell, Nina Pitre, Aura Rexach, Kiley Sheehan, Laura E. Williams

**Affiliations:** a Department of Biology, Providence College, Providence, Rhode Island, USA; University of Maryland School of Medicine

## Abstract

After isolating *Pantoea* sp. strain SOD02 from an urban freshwater stream in Providence, RI, we used PacBio RSII data for *de novo* assembly and Illumina MiSeq data for polishing. This yielded complete circular sequences for a 4,227,027-bp chromosome with 54.7% GC and a 926,844-bp plasmid with 54.0% GC.

## ANNOUNCEMENT

To obtain bacteria for use as prey in our studies of predatory *Bdellovibrio* (1), we swabbed water from an urban stream in Providence, RI (41.835°N, 71.443°W), onto Trypticase soy agar and incubated at 28°C. After three rounds of picking and streaking, we grew pure culture overnight in Trypticase soy broth (TSB) at 28°C and then combined the culture 1:1 with 50% glycerol to establish freezer stocks.

We used the Wizard genomic DNA purification kit (Promega, Madison, WI) to extract DNA from separate overnight cultures grown from freezer stock in TSB at 28°C. The University of Maryland Institute for Genome Sciences used one extraction for long-read sequencing, which involved shearing DNA using a g-TUBE at 3,400 rpm, size selection on a Blue Pippin instrument with an 11,000-bp cutoff, library preparation using SMRTbell template prep kit 1.0, and sequencing one SMRT (single-molecule real-time) cell on PacBio RS II with P6-C4 chemistry. The University of Rhode Island Genomics and Sequencing Center used another extraction for short-read sequencing, which involved shearing DNA using a Covaris S220 focused ultrasonicator, library preparation using PrepX DNA library kit, visualization on high-sensitivity BioAnalyzer chips, quantification with the KAPA Illumina quantification kit, and sequencing on Illumina MiSeq to obtain 2× 250-bp paired-end reads.

Unless otherwise noted, default parameters were used for all software. We compared Hierarchical Genome Assembly Process v3 (HGAP3) (2) and Canu 2.2 (3) for *de novo* assembly of PacBio data (119,255 subreads at an *N*_50_ of 13,141 bp). We tested HGAP3 with estimated genome size 4.5 Mbp, which generated two contigs. After identifying and trimming overlap between contig ends with BLASTN (4) and EMBOSS 6.6.0.0 (5) extractseq, the circularized contigs were 4,226,901 and 926,802 bp. We tested Canu with an estimated genome size of 4.25 Mbp, which generated two contigs. After trimming overlap with extractseq based on Canu information, the circularized contigs were 4,226,861 and 926,798 bp. Assemblies were almost identical by dnadiff (6), with 198 indel bp between chromosome contigs and one single nucleotide variant (SNV) and 84 indel bp between plasmid contigs. To proceed, we used the circularized contigs from Canu and rotated them to start at *dnaA* for the chromosome and *repB* for the plasmid.

For polishing, we separately aligned raw Illumina MiSeq read 1 (R1) and read 2 (R2) datasets (5,181,180 reads each) to the circularized and rotated contigs using the Burrows-Wheeler aligner “mem” (BWA-mem) 0.7.17 (7) with the option to report all possible alignments for each read. Samtools (8) analysis showed 99.3% R1 and 97.8% R2 reads aligned to at least one location. Using Polypolish v0.5.0 (9), we removed alignments based on insert size and then corrected the sequence. For the chromosome, Polypolish reported 437× coverage and corrected 168 indel bp. For the plasmid, Polypolish reported 375× coverage and corrected 48 indel bp. To confirm, we aligned MiSeq reads to corrected contigs using POLCA within MaSuRCA 4.0.8 (10), which made no additional corrections. The chromosome is 4,227,027 bp (54.7% GC) with 3,816 protein-coding genes, 78 tRNAs, and 22 rRNAs predicted by annotation with PGAP version 6.2 (11). The plasmid is 926,844 bp (54.0% GC) with 796 protein-coding genes. Digital DNA:DNA hybridization analysis using the Type Strain Genome Server (12) classified SOD02 in the bacterial genus *Pantoea*.

### Data availability.

*Pantoea* sp. strain SOD02 sequences have been deposited in GenBank under chromosome no. CP102604 and plasmid no. CP102605. PacBio and MiSeq reads have been deposited in the SRA under BioProject no. PRJNA866139 and SRA no. SRX16926094 and SRX16926095, respectively.
